# Association of Duffy Blood Group Gene Polymorphisms with *IL8* Gene in Chronic Periodontitis

**DOI:** 10.1371/journal.pone.0083286

**Published:** 2013-12-26

**Authors:** Emília Ângela Sippert, Cléverson de Oliveira e Silva, Jeane Eliete Laguila Visentainer, Ana Maria Sell

**Affiliations:** 1 Post Graduation Program of Biosciences Applied to Pharmacy, Department of Analysis Clinical and Biomedicine, Maringa State University, Parana, Brazil; 2 Dentistry Department, Maringa State University, Parana, Brazil; 3 Basic Health Sciences Department, Maringa State University, Parana, Brazil; Instituto de Higiene e Medicina Tropical, Portugal

## Abstract

The antigens of the Duffy blood group system (DARC) act as a receptor for the interleukin IL-8. IL-8 plays an important role in the pathogenesis of chronic periodontitis due to its chemotactic properties on neutrophils. The aim of this study was to investigate a possible association of Duffy blood group gene polymorphisms with the *-353T>A, -845T>C* and *-738T>A* SNPs of the *IL8* gene in chronic periodontitis. One hundred and twenty-four individuals with chronic periodontitis and 187 controls were enrolled. DNA was extracted using the salting-out method. The Duffy genotypes and *IL8* gene promoter polymorphisms were investigated by PCR-RFLP. Statistical analyses were conducted using the Chi square test with Yates correction or Fisher's Exact Test, and the possibility of associations were evaluated by odds ratio with a 95% confidence interval. When analyzed separately, for the Duffy blood group system, differences in the genotype and allele frequencies were not observed between all the groups analyzed; and, in nonsmokers, the *-845C* allele (3.6% vs. 0.4%), *-845TC* genotype (7.3% vs. 0.7%) and the CTA haplotype (3.6% vs. 0.4%) were positively associated with chronic periodontitis. For the first time to our knowledge, the polymorphisms of erythroid DARC plus *IL8 -353T*>*A* SNPs were associated with chronic periodontitis in Brazilian individuals. In Afro-Brazilians patients, the FY*02N.01 with *IL8 -353A* SNP was associated with protection to chronic periodontitis.

## Introduction

The Duffy blood group system is composed of five antigens, also called the Duffy antigen receptor chemokines (DARC), which occur as membrane glycoproteins [1-3]. These antigens are present on red blood cells (RBCs) and endothelial cells lining the post-capillary venules in most tissues, except the brain, where the Duffy antigen is expressed in Purkinje cells [4].

Besides being a receptor of the *Plasmodium vivax* and *knowlesi* malarial parasites [5], HIV-1 [6] and molecules such as tetraspanin CD82 [7], DARC is also a high-affinity receptor for the CXC class of pro-inflammatory chemokines, such as IL-8, and CC, including monocyte chemoattractant protein-1 (MCP-1), macrophage inflammatory protein-1 (MIP) and regulated and normal T cell expressed and secreted (RANTES) [8]. Unlike typical chemokine receptors, DARC does not activate the intracellular signaling cascade linked to protein G, a key event in the signaling of cell migration, because it lacks the highly conserved Asp-Arg-Tyr (DRY) motif in the second intracellular loop of the protein, and is therefore considered a silent receptor [9,10].

The main antigens of the Duffy blood group system, Fy^a^ and Fy^b^, are encoded by two allele forms of the *FY* gene, the FY*01 and FY*02 alleles respectively, which differ by a single base substitution at nucleotide 125 [11]. This substitution of an amino acid in the amino-terminal domain of the protein does not affect the binding affinity of chemokines [12]. Most West Africans and two-thirds of Afro-Americans do not express Fy^a^ or Fy^b^ on the surface of RBCs (the Fy(a−b−) phenotype) due to a homozygous nucleotide base change in the 5´ untranslated region of the *FY* gene, -67T>C, which is also called the GATA-1 box mutation. The genotype found in these individuals is FY*02N.01/FY*02N.*01* [11,13-15]. The rare allele, FY*02M.01, characteristic of Caucasians, encodes a weak expression of Fy^b^ in RBCs due to two polymorphisms in exon 2 of the *FY* gene (265C>T and 298G>A) [16]. In Brazil, due to the miscegenation of Afro-Brazilians, Brazilian Indians and Europeans, these polymorphisms are also found in other ethnic groups [17].

Recent studies have shown that the adsorption of IL-8 onto RBCs by DARC leads to an increased recruitment of leukocytes from the blood to the tissue compared to individuals without DARC on the RBCs. This is because DARC removes the "desensitizing" plasma chemokines (free circulating IL-8 binds to neutrophils and prevents their chemotaxis) and indirectly improves the coding signal of IL-8 on the surface of endothelial cells (*via* immobilization by glycosaminoglycans) at predestined sites to recruit neutrophils [18].

IL-8 is responsible for inducing chemotaxis and mediates the activation and migration of neutrophils into tissues from a peripheral blood [18]. The *IL8* gene is located on chromosome 4q13-q21 (GeneBank accession number M28130) and more than 70 single nucleotide polymorphism (SNPs) have been identified. The expression levels of a protein may be modulated by genetic polymorphisms in regulatory regions of the gene, mainly the promoter regions, and the *IL8 -353 T>A* (rs4073) SNP has been shown to regulate IL-8 levels after stimulation with lipopolysaccharide (LPS) [19]: IL-8 production was highest to AA group, least to TT group and intermediated to AT group at same time. Furthermore, *-353 T>A* *-738 T>A* and *-845 T>C* formed a haplotype in the *IL8* gene [20] and linkage disequilibrium was observed between the -845 and -738 SNPs and -738 and -353 SNPs [21].

Due to its chemotactic properties on neutrophils, IL-8 plays an important role in the pathogenesis of chronic periodontitis [22], a disease characterized by a destructive inflammatory process that affects the tissues of the tooth.

Polymorphisms of the *FY* gene may lead to reduced or even absent DARC expression on RBCs, while *IL8* single nucleotide polymorphisms (SNPs) affect IL-8 production. Thus, examining these polymorphisms may contribute to the understanding of the immunopathogenesis underlying chronic periodontitis.

## Methods

### Sample selection

This study involved individuals from the north and northwest regions of the state of Paraná, southern Brazil. A total of 311 individuals were selected from those who sought dental treatment in the dental clinics of the Maringa State University (UEM) and Dental School of Inga (UNINGÁ) from January 2012 to September 2012. Males and females aged over 18 years and from all ethnic groups and with at least 20 teeth in the buccal cavity participated in this study. Individuals with acute infections or lactations and those who were pregnant were not included. After taking the patient’s history, clinical periodontal examinations were conducted by two examiners. Clinical parameters of probing depth and clinical insertion level were examined at six sites (mesiovestibular, vestibular, distovestibular, mesiolingual, lingual and dentilingual) of each tooth, as was bleeding on probing. After the periodontal examination, participants were categorized into two different groups: the chronic periodontitis group (n = 124) composed of individuals who had at least 5 sites in different teeth with periodontal pockets larger than or equal to 5 mm and clinical insertion level values greater than or equal to 3 mm; and the control group (n = 187), formed by individuals who did not have sites with reduced clinical insertion level values, displayed a probing depth of less than 4 mm and exhibited no bleeding on probing.

Information on the patient’s ethnic background, presence of other diseases such as diabetes, and smoking were obtained by interviewing the individual (anamnesis).

### Ethics Information

All individuals who agreed to participate in this research were informed about the nature of the study and signed an informed consent form. This study was approved by the Human Research Ethics Committee of the Maringa State University (UEM - No. 719/2011, 02/12/2011).

### Sample collection and DNA extraction

To extract the DNA, the buffy coat was obtained from the 4 mL of peripheral blood collected in EDTA by centrifugation (210 *g* for 15 min). The DNA was extracted using the salting-out method. The concentration and quality of the DNA were analyzed by optical density in a Thermo Scientific Nanodrop 2000^®^ apparatus (Wilmington, USA).

### Genotyping of the Duffy blood group system

The *FY* polymorphisms, including the SNPs 125 G>A (FY*01/FY*02), 265 C>T, 298 G>A (FY*02M.01) and the GATA-1 box mutation −67T>C (FY*02N.01), were determined by polymerase chain reaction–restriction fragment length polymorphism (PCR–RFLP), as previously described by Castilho et al. [23], and described in the Table 1. 

**Table 1:** PCR-RFLP: primers, cycling conditions and enzymes used to the genotyping of the Duffy blood group system.

SNP	Primer sequence (GenBank: S76830.1)	Restriction enzyme	Fragments after enzymatic digestion (bp)
*125G>A (rs12075)	5′-tccccctcaactgagaactc-3′	BanI	(G) 86 + 96 + 210
	5′-catggcaccgtttggttcag-3′		(A) 86+ 306
*265C>T (rs34599082)	5′-tccccctcaactgagaactc-3′	MspAI	(C) 85 + 119
	5′-catggcaccgtttggttcag-3′		(T) 85 + 119 + 161
*298G>A (rs13962)	5′-tccccctcaactgagaactc-3′	MwoI	(G) 86 + 96 + 210
	5′-catggcaccgtttggttcag-3′		(A) 86+ 306
^**^-67T>C (rs2814778)	5′-caacatctgtgtaccctg-3′	StyI	(T) 81 + 108
	5′-catggcaccgtttggttcag-3′		(C) 61 + 81 + 108

Cycling conditions: * 95°C, 15’; 35 cycles (94°C, 20”; 62°C, 20”; 72°C, 20”) and 72°C, 10’

** 95°C, 15’; 35 cycles (94°C, 20”; 62°C, 20”; 72°C, 20”) and 72°C, 10’

### Analysis of IL8 promoter polymorphisms

Polymorphisms of the *IL8* promoter region were investigated by PCR-RFLP: the -845T>C and -353T>A SNPs were studied using an adapted procedure of Rovin et al. [20], and the -738T>A SNP was determined using the method of Heinzmann et al. [24] and Lee et al. [25], described in the Table 2.

**Table 2:** PCR-RFLP: primers, cycling conditions and enzymes used to identify polymorphisms in the *IL8* promoter region.

SNP	Primer sequence (GenBank:28130)	Restriction enzyme	Fragments after enzymatic digestion (bp)
^*^-845T>C (rs2227532)	5′-gaattcagtaacccaggcat-3′	AseI	(T) 791 + 736
	5′- aagcttgtgtgctctgctgtc-3′		(C) 1527
^*^-353T>A (rs4073)	5′-gaattcagtaacccaggcat-3′	MfeI	(A) 1230 + 297
	5′- aagcttgtgtgctctgctgtc-3′		(T) 1527
^**^-738T>A (^***^)	5′-aacccagcagctccagtg-3′	XbaI	(T) 302 + 232
	5′-agataagccagccaatcatt-3′		(A) 534

bp: base pairs;

Cycling conditions: * 95°C, 3’; 35 cycles (95°C 45”; 56°C, 30”; 68°C, 2’) and 68°C, 8’

** 94°C, 5’; 35 cycles (94°C, 30”; 61°C, 1’; 72°C, 1’) and 72°C, 8’

(***) It was not found a rs (reference sequence) identification number related to this SNP;

### Statistical analysis

Allele and genotype frequencies were obtained by direct counts. The data were tested for their fit to Hardy-Weinberg equilibrium by calculating the expected frequencies of the genotypes and comparing them with the observed values, to test the deviation of genotype distribution. The Arlequin software, version 3.1 [26] was used to investigate linkage disequilibrium and the estimated frequencies of the possible haplotypes of *IL8*. The association between genetic polymorphisms and chronic periodontitis was evaluated using the Chi-square test with Yates correction or the Fisher's exact test for sample sizes of less than five. The correlation was deemed present by an odds ratio with 95% confidence intervals only for significant p-values. For these analyses, OpenEpi program Version 2.3.1 was used [27]. All tests were carried out using a significance level of 5%. 

## Results

The study population consisted of 311 unrelated individuals: 124 patients with chronic periodontitis and 187 controls. Most participants were female (60.5%), nonsmokers (60.8%) and Caucasian (64.3% - Table 3). Diabetes was significantly more frequent in patients than in controls, confirming its association to chronic periodontitis (p-value < 0.001; OR = 3.313; 95% CI = 1.883 -5.882). Smoking was also an important risk factor: smokers and ex-smokers were between 2 and 3 times more likely to develop chronic periodontitis than nonsmokers (Table 3). To exclude smoking as a predisposing factor, statistical analyses were performed in the total sample (smokers and nonsmokers), as well as in nonsmokers. The periodontitis patients that had diabetes were included in total sample and nonsmokers groups. However, although they are present in small numbers, these subjects were excluded from the groups during statistical analysis to prevent diabetes as predisposing factor. The genotype distributions of each SNP studied were consistent with the assumption of the Hardy–Weinberg equilibrium in the control and periodontal disease groups (p-value >0.05). 

**Table 3:** Characteristics of patients with chronic periodontitis and control population.

**Category**	**Subcategory**	**Control**	**Periodontitis**	**Total**
		**N = 187**	**N = 124**	(%)
		n (%)	n (%)	n (%)
Gender	Female	120 (64.2)	68 (54.8)	188 (60.5)
	Male	67 (35.8)	56 (45.2)	123 (39.5)
Age	18 - 33 years	35 (18.7)	16 (12.9)	51 (16.4)
	34 - 49 years	98 (52.4)	65 (52.4)	163 (52.4)
	50 - 65 years	50 (26.7)	35 (28.2)	85 (27.3)
	66 - 81 years	4 (2.1)	8 (6.5)	12 (3.9)
Ethnic background	Caucasian	128 (68.5)	72 (58.1)	200 (64.3)
	Afro-Brazilian	14 (7.5)	15 (12.1)	29 (9.3)
	Mulatto	45 (24.1)	37 (29.8)	82 (26.3)
Smoking	Nonsmoker^*^	134 (71.7)	55 (44.4)	189 (60.8)
	Smoker^**^	23 (12.3)	28 (22.6)	51 (16.4)
	Ex- smoker^***^	30 (16.0)	41 (33.1)	71 (22.8)
Diabetes	No	180 (96.3)	109 (87.9)	289 (92.9)
	Yes^****^	7 (3.7)	15 (12.1)	22 (7.1)

* p-value < 0.001; OR = 0.31; 95% CI = 0.19-0.50;

** p-value = 0.03; OR = 2.07; 95% CI = 1.12-3.84;

*** p-value < 0.001; OR = 2.95; 95% CI = 1.56-5.62;

p-value < 0.001; OR = 3.31; 95% CI = 1.88-5.88.

For the Duffy blood group system, the prevalent genotype in all the analyzed groups was FY*01/FY*02, with FY*02 being the most common allele (Table 4). Differences in the genotype and allele frequencies were not observed between all the groups analyzed.

**Table 4:** Distribution of genotypes and alleles of the Duffy blood group system in patients with chronic periodontitis and controls.

**Duffy**	**Total**		**Nonsmokers**	
	**Periodontitis**	**Controls**	**Periodontitis**	**Control**
	**N = 124**	**N = 187**	**N = 55**	**N = 134**
	n(%)	n (%)	n(%)	n (%)
**Genotypes**				
FY*01/FY*01	10 (8.1)	27 (14.4)	10 (18.1)	23 (17.2)
FY*01/FY*02	45 (36.3)	62 (33.2)	15 (27.3)	41 (30.6)
FY*02/FY*02	33 (26.6)	57 (30.5)	13 (23.6)	40 (29.9)
FY*02/FY*02N.01	10 (8.1)	12 (6.4)	4 (7.3)	9 (6.7)
FY*02/FY*02M.01	2 (1.6)	3 (1.6)	1 (1.8)	2 (1.5)
FY*01/FY*02N.01	12 (9.7)	10 (5.3)	6 (11)	8 (6)
FY*02N.01/FY*02N.01	7 (5.6)	11 (5.9)	3 (5.5)	9 (6.7)
FY*02M.01/FY*02N.01	2 (1.6)	3 (1.6)	1 (1.8)	1 (0.7)
FY*01/FY*02M.01	3 (2.4)	2 (1.1)	2 (3.6)	1 (0.7)
**Alleles**				
FY*01	80 (32.3)	128 (34.2)	43 (39.1)	96 (35.8)
FY*02	123 (49.6)	191 (51.1)	46 (41.8)	132 (49.3)
FY*02N. 01	38 (15.3)	47 (12.6)	17 (15.5)	36 (13.4)
FY*02M.01	7 (2.8)	8 (2.1)	4 (3.6)	4 (1.5)

The genotype and allele frequencies of the *-845T>C*, *-738T>A* and *-353T>A* SNPs in the *IL8* gene promoter region are listed in Table 5. The *-845C* allele was most common in non-smoking patients with chronic periodontitis compared to controls (3.6% *vs* 0.4%; p-value = 0.024; OR = 10.4; 95% CI = 1.1-91.2), as was the *-845 TC* genotype (7.3% *vs* 0.7%; p-value = 0.024; OR = 10.1; 95% CI = 1.1-95.5) and the CTA haplotype (3.6% *vs* 0.4%; p-value = 0.024; OR = 10.1; 95% CI = 1.1-91.2), indicating positive correlations. Moreover, the CTA/TTT genotype was present only in patients with periodontitis (5.5% *vs* 0%; p-value = 0.024).

**Table 5:** Allele, genotypic and haplotype frequencies of the -845T>C, -738T>A and -353T>A polymorphisms in the *IL8* promoter region in patients with periodontitis and controls.

***IL8* SNPs**	**Total**		**Nonsmokers**	
	**Periodontitis**	**Controls**	**Periodontitis**	**Controls**
	**N = 124**	**N = 187**	**N = 55**	**N = 134**
	n (%)	n (%)	n (%)	n (%)
***-845***				
*T*	240 (96.8)	370 (98.9)	106 (96.4) **^*a*^**	267 (99.6) **^*a*^**
*C*	8 (3.2)	4 (1.1)	4 (3.6) **^*b*^**	1 (0.4) **^*b*^**
*TT*	117 (94.4)	183 (97.9)	51 (92.7) **^*c*^**	133 (99.3) **^*c*^**
*TC*	6 (4.8)	4 (2.1)	4 (7.3) **^*d*^**	1 (0.7) **^*d*^**
*CC*	1 (0.8)	0 (0)	0 (0)	0 (0)
***-738***				
*T*	247 (99.6)	373 (99.7)	109 (99.1)	267 (99.6)
*A*	1 (0.4)	1 (0.3)	1 (0.9)	1 (0.4)
*TT*	123 (99.2)	186 (99.5)	54 (98.2)	133 (99.3)
*TA*	1 (0.8)	1 (0.5)	1 (1.8)	1 (0.7)
***-353***				
*T*	130 (52.4)	198 (52.9)	63 (57.3)	145 (54.1)
*A*	118 (47.6)	176 (47.1)	47 (42.7)	123 (45.9)
*TT*	34 (27.4)	53 (28.3)	17 (30.9)	38 (28.4)
*TA*	62 (50)	92 (49.2)	29 (52.7)	69 (51.5)
*AA*	28 (22.6)	42 (22.5)	9 (16.4)	27 (20.1)
***-845-738-353***				
*TTT*	131 (52.8)	198 (53)	63 (57.3)	145 (54.1)
*TTA*	108 (43.6)	171 (45.7)	42 (38.2)	121 (45.1)
*TAA*	1 (0.4)	1 (0.3)	1 (0.9)	1 (0.4)
*CTA*	8 (3.2)	4 (1.0)	4 (3.6) **^*e*^**	1 (0.4) **^*e*^**
***-845-738-353/845-738-353***				
*TTT/TTA*	57 (46)	89 (47.6)	25 (45.5)	68 (50.7)
*TTT/TTT*	34 (27.4)	53 (28.3)	17 (30.9)	38 (28.4)
*TTA/TTA*	25 (20.2)	40 (21.4)	8 (14.5)	26 (19.4)
*TTT/TAA*	1 (0.8)	1 (0.5)	1 (1.8)	1 (0.75)
*TTA/CTA*	2 (1.6)	2 (1.1)	1 (1.8)	1 (0.75)
*CTA/TTT*	4 (3.2)	2 (1.1)	3 (5.5) **^*f*^**	0 (0) **^*f*^**
*CTA/CTA*	1 (0.8)	0 (0)	0 (0)	0(0)

^a^ p-value= 0.027; OR=0.1; CI95%=0.01- 0.9;

^b^ p-value=0.024; OR=10.4; CI95%=1.1-91.2;

^c^ p-value=0.027; OR=0.1; CI95%=0.01- 0.9;

^d^ p-value=0.024; OR=10.1; CI95%=1.1-95.5;

^e^ p-value=0.024; OR=10.1; CI95%=1.1-91.2;

^f^ p-value= 0.024; OR, IC undefined (because the presence of zero).

To evaluate DARC expression on the surface of RBCs, two new groups were formed: the first, without polymorphisms, was formed by grouping individuals with the FY*01 and FY*02 alleles encoding Fy^a^ and Fy^b^ expression, respectively, and the other, with polymorphisms, was composed of individuals carrying the *FY* alleles with the *-67T>C* SNP, FY*02N.01 allele, and the *265T*>*C* SNP, FY*02M.01 allele. As the frequencies of DARC polymorphisms vary in different ethnic groups, the analyses were performed separately for Afro-Brazilians and Admixed-Brazilians. Brazilians population is very mixed and, in the Parana State, the degree of the European ancestry is high (80.6%), with a small but significant contribution of African (12.5%) and Amerindian (7.0%) genes, according to Probst et al. [28]. Thus the studied populations were considered as Admixed-Brazilians (Mulattos or *pardos* plus whites individuals) and Afro-Brazilians (black individuals). The results are shown in Table 6. 

**Table 6:** Distribution of *FY* allele frequencies with and without the -67T>C (FY*02N.01) and -265T>C (FY*02M.01) SNPs in Afro-Brazilian and Admixed-Brazilian patients with chronic periodontitis and controls.

**Duffy**	**Periodontitis**	**Controls**
	n (%)	n (%)
**Afro-Brazilians**	**N = 15**	**N = 14**
FY*01 and FY*02	20 (66.7)	12 (42.8)
FY*02N.01	10 (33.3)	16 (57.2)
**Admixed Brazilians**	**N = 109**	**N = 173**
FY*01 and FY*02	183 (83.9)	307 (88.7)
FY*02N.01 and FY*02M.01	35 (16.1)	39 (11.3)

The distribution of frequencies of the *FY* alleles without and with polymorphisms was similar between patients and controls. However, differences, although statistically non-significant, were observed in Afro-Brazilians. Afro-Brazilian patients with chronic periodontitis displayed a lower frequency of *FY* alleles with polymorphism (33.3% *vs* 57.2%). The allele, genotype and haplotype frequencies of the *-845T>C*, *-738T>A* and *-353T>A* polymorphisms of *IL8* were analyzed separately in patients with periodontitis and controls, and in Afro-Brazilians and Admixed-Brazilians (Table 7). The frequency of the *-353A* SNP (allele and genotype) was lower in Afro-Brazilian patients (40% *vs* 60.7%), although not significantly. Moreover, the TTA haplotype had a lower frequency in Afro-Brazilians (33.3% *vs* 60.7%; p-value = 0.067; OR = 0.3; 95%CI = 0.1-0.9).

**Table 7:** Allele, genotype and haplotype frequencies of the *-845T>C*, *-738T>A* and *-353T>A* polymorphisms in the *IL8* promoter region in Afro-Brazilian and Admixed-Brazilians patients and controls.

**SNP**	**Afro-Brazilians**		**Admixed Brazilians**	
	**Periodontitis**	**Controls**	**Periodontitis**	**Controls**
	**N = 15**	**N = 14**	**N = 109**	**N = 173**
	n (%)	n (%)	n (%)	n (%)
***-845***				
***T***	28 (93.3)	28 (100)	212 (97.2)	342 (98.8)
***C***	2 (6.7)	0 (0)	6 (2.8)	4 (1.2)
***TT***	13 (86.7)	14 (100)	104 (95.4)	169 (97.7)
***TC***	2 (13.3)	0 (0)	4 (3.7)	4 (2.3)
***-738***				
***T***	30 (100)	28 (100)	217 (99.5)	345 (99.7)
***A***	0 (0)	0 (0)	1 (0.5)	1 (0.3)
***TT***	15 (100)	14 (100)	108 (99.1)	172 (99.4)
***TA***	0 (0)	0 (0)	1 (0.9)	1 (0.6)
***-353***				
***T***	18 (60.0)	11 (39.3)	112 (51.4)	187 (54.0)
***A***	12 (40.0)	17 (60.7)	106 (48.6)	159(46.0)
***TT***	5 (33.3)	3 (21.4)	29 (26.6)	50 (28.9)
***TA***	8 (53.3)	5 (35.7)	54 (49.5)	87 (50.3)
***AA***	2 (13.3)	6 (42.9)	26 (23.9)	36 (20.8)
***-845-738-353***				
***TTT***	18 (60)	11 (39.3)	113 (51.8)	187 (54.0)
***TTA***	10 (33.3)^*^	17 (60.7)^*^	98 (45.0)	154 (44.5)
***TAA***	0 (0)	0 (0)	1 (0.5)	1 (0.3)
***CTA***	2 (6.7)	0 (0)	6 (2.8)	4(1.2)
***-845-738-353/-845-738-353***				
***TTT/TTA***	6 (40.0)	5 (35.7)	51 (46.8)	84 (48.6)
***TTT/TTT***	5 (33.4)	3 (21.4)	29 (26.6)	50 (28.9)
***TTA/TTA***	2 (13.3)	6 (42.9)	23 (21.1)	34 (19.7)
***TTT/TAA***	0 (0)	0 (0)	1 (0.9)	1 (0.6)
***TTA/CTA***	0 (0)	0 (0)	2 (1.8)	2 (1.2)
***CTA/TTT***	2 (13.3)	0 (0)	2 (1.8)	2 (1.2)

* p-value = 0.067; OR = 0.3; 95% CI = 0.1- 0.9.

The genotypes -845CC, -738AA and TAT, CTT, CAT, CAA and CTA/CTA were not found in patients and controls.

An analysis of the *FY* genotypes both with and without polymorphism *versus* the *-353T>A IL8* polymorphism was performed in patients and controls (Table 8). In Afro-Brazilians patients, the presence of the *FY* genotype with polymorphism and the *-353A* SNP was lower (20% *vs* 50%; p-value = 0.032; OR = 0.25; 95% CI = 0.078-0.79). The genotype *-353AA* was also lower in the same group, although not significant (6.7% *vs* 35.7%; p-value= 0.06). In Admixed-Brazilians, the simultaneous presence of the *FY* genotype the *-353T>A IL8* polymorphism was not significant.

**Table 8:** Distribution of *FY* genotypes with the presence and absence of the *-67T>C* and 265*T*>*C* polymorphisms versus the *-353T>A* *IL8* SNP in patients with chronic periodontitis and controls.

	**Periodontitis**					**Controls**				
	**-353 *IL-8***					**-353 *IL-8***				
**Duffy genotypes**	TT	TA	AA	A	T	TT	TA	AA	A	T
**Total**	**N = 124**					**N = 187**				
*Without polymorphism^a^*	24 (19.4)	42 (33.9)	22 (17.7)	86 (34.7)	90 (36.3)	43 (23)	72 (38.5)	31 (16.6)	134 (35.8)	158 (42.2)
*With polymorphism^b^*	10 (8.1)	20 (16.1)	6 (4.8)	32 (12.9)	40 (16.1)	10 (5.34)	21 (11.22)	10 (5.34)	41 (11)	41 (11)
**Afro-Brazilians**	**N = 15**					**N = 14**				
*Without polymorphism*	3 (20)	4 (26.7)	1 (6.7)	6 (20)	10 (33.3)	2 (14.3)	1 (7.1)	1 (7.1)	3 (10.7)	5 (17.9)
*With polymorphism*	2 (13.3)	4 (26.7)	1 (6.7)	6 (20)^c^	8 (26.7)	1 (7.1)	4 (28.6)	5 (35.7)	14 (50)^c^	6 (21.4)
**Admixed- Brazilians**	**N = 109**					**N = 173**				
*Without polymorphism*	21 (19.3)	38 (34.8)	21 (19.3)	80 (36.7)	80 (36.7)	41 (23.7)	71 (41.0)	30 (17.3)	131 (37.9)	153 (44.2)
*With polymorphism*	8 (7.3)	16 (14.7)	5 (4.6)	26 (11.9)	32 (14.7)	9 (5.2)	16 (9.2)	6 (3.5)	28 (8.1)	34 (9.8)

^a^ *FY* genotypes without *-67T>C* and/or 265*T*>*C* SNPs;

^b^ *FY* genotypes with *-67T>C* and/or 265*T*>*C* SNPs in homozygosis or heterozygosis (without DARC expression or lower DARC expression);

^c^p-value = 0.03; OR = 0.25; 95% CI = 0.078- 0.79;

## Discussion

The Duffy blood group system acts as a receptor for IL-8, an inflammatory chemokine involved in neutrophil activation and trafficking [10,29-31]. When IL-8 is bound to DARC on the surface of RBCs, it is effectively inactivated. RBCs of individuals with the Fy(a‑b-) phenotype do not bind IL-8 and therefore, do not have this function of sink as proposed for the DARC on RBCs [10,29]. As high levels of serum IL-8 and neutrophils are involved in the pathogenesis of chronic periodontitis [32], we decided to investigate the role of DARC in this disease.

Several periodontal disease risk factors have been previously identified [33,34], including diabetes, a strong causal risk factor for periodontal pathology [35-39]. In the present study, subjects with diabetes were three times more likely to develop chronic periodontitis than non-diabetic subjects (p-value <0.001; OR = 3.31; 95%CI = 1.88-5.88). The biological plausibility has been well documented. The potential influence of diabetes on periodontal disease is likely to be due to the hyperinflammatory response to infection, uncoupling of bone destruction and repair, and/or the effects of advanced glycation and the resulting products [38,39]. The proinflammatory cytokine profiles in the diabetes mellitus patients, including IL-8, were correlated with severity of the course of disease [40-43] and polymorphisms of pro-inflammatory cytokine genes (*CCL2, TGFB1, IL8, CCR5*, and *MMP9*) were found to be associated with the risk of diabetic nephropathy [44]. Although the diabetes patients were present in small numbers, these subjects were excluded from the groups during statistical analyses of *IL8* SNPs (dates not shown) and no differences were observed.

Smoking is another important risk factor for the onset and progression of chronic periodontitis [45-47] and the present study confirmed these findings. Potential mechanisms for the effect of smoking on periodontal disease include immunosuppression and exacerbated inflammatory cell response [48]. The risk associated with smoking can often obscure genetic risk factors [49]; thus, the statistical analysis of the subgroup of nonsmokers was carried out to exclude any possible influence of smoking on the genetic factors.

The distribution of *FY* alleles and genotypes and most of the *IL8* polymorphisms *-845T*>*C*, *-738T>A* and *-353T>A* were similar between patients with chronic periodontitis and controls (total population and nonsmokers). The exception was the *-845T>C IL8* polymorphism in nonsmokers: the *-845C* allele and *-845TC* genotype were associated with susceptibility to chronic periodontitis (*-845C* allele: 3.6% *vs* 0.4%; OR = 10.4; *-845TC* genotype: 7.3% *vs* 0.7%; OR = 10.1). Thus, individuals who had the *-845C* allele or *-845TC* genotype were 10 times more likely to develop the disease than those who did not. As far as we know, the IL8 -845*T*>*C* and *-738T>A* SNPs are not associated with different levels of production of this cytokine. Moreover, these SNPs have been associated with disease in only a few studies [20,21,50], possibly due to low genetic diversity: C and A mutant alleles were observed in Afro-American populations (C: 8% and A: 5%) [20], but were absent in Europeans [20,21]. Kim et al. [21] studied the association of the *-845T>C*, *-738T>A* and *-353T>A* *IL8* polymorphisms in chronic periodontitis in a Brazilian population and did not observe any association between alleles and genotypes in the total sample or in nonsmokers. However, in the haplotype analysis, significant differences were obtained for the CTA, TTA and TAT haplotypes, which were associated with susceptibility to periodontitis and TTT and TAA seemed to have the opposite effects, in the total sample and in nonsmokers. Nonsmokers carrying the TAT/CTA and TTA/CAT genotype seemed to be susceptible and those that carrying TTT/TAA seemed protected against the development of chronic periodontitis. In the current study, the CTA haplotype and CTA/TTT genotype were associated with susceptibility to the disease. However, TAT was not found because the frequency of the *-738A* allele in the study population was very low (<1%). CTA/TTT genotype was only found in patients with chronic periodontitis (5% *vs* 0%; p-value = 0.024).

The *IL8 -353TγA* SNP, also found in the literature as *-251T>A* and *rs4073TA*, was linked to high transcription levels of IL-8 after whole blood stimulation with lipopolysaccharides [19]. This polymorphism has been described in the literature as being associated with various diseases: respiratory syncytial virus bronchiolitis, prostate cancer, distal gastric cancer, breast cancer, oral squamous cell carcinoma, age-related macular degeneration and oral lichen planus [19,50-55]. In Brazil, studies correlating the *IL8 -353TγA* SNP with chronic periodontitis were carried out by Kim et al. in 2009 [56] and Andia et al. in 2011 [57]. Kim et al. evaluated the population as a whole, as well as nonsmokers, and found no association with periodontitis. Andia et al., who only studied nonsmokers, found a reduction in the frequency of the TT genotype (13.8% *vs* 35.2%) and a significant increase in the frequency of the TA genotype (74.6% *vs* 52.8%) associated with periodontitis; and susceptibility to periodontitis in individuals with the TA genotype was also observed when Caucasians were analyzed separately. High levels of IL-8 mRNA were also identified in individuals with the TA genotype. The differences of these results may be related to the selection of groups (smokers or nonsmokers) and the differences in the composition of the population, given that the Brazilian population is highly racially mixed. According to Ioannidis et al. [58], genetic associations may not be uniform across the population and the frequency of different polymorphisms may vary between ethnic groups.

As ethnic background is an important factor to be considered and many studies have reported contradictory results when comparing distinct populations [59-61], we subdivided the study population by two ethnic groups.

When DARC expression on the surface of RBCs was compared between the without and with polymorphism groups of different ethnicities (Afro-Brazilians and Admixed-Brazilians), there were no statistically significant differences, although there was a decrease in the frequency of the *-67T>C* SNP (FY*02N.01 allele) in Afro-Brazilian patients (33% *vs* 57.2%). This decrease may suggest protection from the disease. The *-67T>C* SNP in the 5´ untranslated region of the *FY* gene, resulting in the absence of Fy^b^ from RBCs and eliciting the Fy(b-) phenotype, does not change the expression of this protein in other tissues [13]. This polymorphism is very common in African descents; approximately 67% of Afro-Brazilians have the Fy(a‑b-) phenotype [62]. In this study, we observed that the frequency of the FY*02N.01 allele was lower than expected for this ethnic group in patients with periodontitis, albeit not significant. An analysis of a larger number of Afro-Brazilians may provide clearer conclusions with respect to the *-67T>C* SNP and chronic periodontitis in this ethnic group.

The analysis of the IL8 -845*T*>*C*, *-738T>A* and *-353T>A* SNPs in the Afro-Brazilian ethnic group and Admixed-Brazilians identified no significant differences in genotype and allele frequencies between controls and patients. The C allele of the -845 SNP was rarely found and the A allele of the -738 SNP was not found in Afro-Brazilians. Thus, the TTA haplotype may protect against periodontitis in Afro-Brazilians, with a p-value tending toward statistical significance (p-value = 0.067; OR = 0.3; 95% CI 0.1-0.9). Oppositely, Kim et al. [21] found that individuals with the TTA haplotype were susceptible to periodontitis; however, the population analyzed in their work was mixed and not categorized according to ethnic group.

Differences were not observed when the *FY* genotypes with and without polymorphism were analyzed together versus the *IL8* -353T>A polymorphism in total of patients. However, Afro-Brazilians who were homozygous or heterozygous for the *-67T>C* SNP in the 5´ untranslated region of the *FY* gene and also had the *IL8 -353A* SNP (high producer of IL-8) were resistant to chronic periodontitis (p-value = 0.032; OR = 0.25; 95% CI = 0.078-0.79). In Admixed Brazilians, the Duffy genotype with the *-67T>C* (absence of DARC) and/or *265T*>*C* (low expression of DARC) SNPs, together with the *IL8 -353T* SNP (low production of IL-8), was not related to periodontitis. 

Usually, a specific combination of polymorphisms in different genes, or a gene polymorphism interacting with environmental factors, can significantly affect the risk of an individual developing a phenotype for certain diseases [63,64]. In Afro-Brazilians the *IL8 -353A* SNP (high producer of IL-8) was associated with higher DARC expression, different than expected to this ethnic group (the *FY -67C* allele frequency was 33%, lower than expected). Possibly, this fact is related a compensatory effect. According Hansell et al. [65] inflammation appears to substantially up-regulate DARC expression; and DARC-bound chemokines are incapable of activating chemokine receptor on leukocytes, whilst those in plasma will be free to interact with blood leukocytes (leading to desensitisation) or become immobilized on endothelial surfaces [18]. Thus, based on the results our hypothesis is that for Afro-Brazilians, the over expression of DARC on the surface of RBCs together with a large production of IL-8 are important in protecting against tissue lesions.

To confirm these findings, and to contribute to a better understanding of the etiology and pathogenesis of chronic periodontal disease, the expression of IL-8 has to be assessed and the largest number of African-Brazilian patients should be analyzed.

## Conclusion

For the first time to our knowledge, the polymorphisms of erythroid DARC plus *IL8 -353T*>*A* SNPs were associated with chronic periodontitis in Brazilian individuals. In Afro-Brazilians patients, the FY*02N.01 with *IL8 -353A* SNP was associated with protection to chronic periodontitis. 
